# Efficacy and acceptability of pharmacological and non-pharmacological interventions for non-specific chronic low back pain: a protocol for a systematic review and network meta-analysis

**DOI:** 10.1186/s13643-020-01398-3

**Published:** 2020-06-05

**Authors:** Trevor Thompson, Sofia Dias, Damian Poulter, Sharon Weldon, Lucy Marsh, Claire Rossato, Jae Il Shin, Joseph Firth, Nicola Veronese, Elena Dragioti, Brendon Stubbs, Marco Solmi, Christopher G. Maher, Andrea Cipriani, John P. A. Ioannidis

**Affiliations:** 1 School of Human Sciences, University of Greenwich, Park Row, London, SE10 9LS UK; 2 Centre for Reviews and Dissemination, University of York, York, UK; 3 School of Health Sciences, University of Greenwich, London, SE9 2UG UK; 4 Barts Health NHS Trust, The Royal London Hospital, Whitechapel Rd, Whitechapel, E1 1BB UK; 5 Department of Pediatrics, Yonsei University College of Medicine, Seoul, Republic of Korea; 6 NICM Health Research Institute, Western Sydney University, Sydney, Australia; 7 Division of Psychology and Mental Health, University of Manchester, Manchester, UK; 8 National Research Council, Neuroscience Institute, Aging Branch, Padova, Italy; 9 Pain and Rehabilitation Centre and Department of Health, Medicine and Caring Sciences, Linköping University, Linköping, Sweden; 10 King’s College London and South London and Maudsley NHS Foundation Trust, London, UK; 11 Neurosciences Department, University of Padua, Padua, Italy; 12 Sydney School of Public Health, The University of Sydney, Sydney, Australia; 13 Department of Psychiatry, University of Oxford, Oxford, UK; 14 Oxford Health NHS Foundation Trust, Warneford Hospital, Oxford, OX3 8AX UK; 15 Meta-Research Innovation Center at Stanford (METRICS) and Departments of Medicine, Health Research and Policy, Biomedical Science and Statistics, Stanford University, Stanford, CA USA

**Keywords:** Low back pain, Network meta-analysis, Systematic review, Protocol, Randomized controlled trial

## Abstract

**Background:**

Despite the enormous financial and humanistic burden of chronic low back pain (CLBP), there is little consensus on what constitutes the best treatment options from a multitude of competing interventions. The objective of this network meta-analysis (NMA) is to determine the relative efficacy and acceptability of primary care treatments for non-specific CLBP, with the overarching aim of providing a comprehensive evidence base for informing treatment decisions.

**Methods:**

We will perform a systematic search to identify randomised controlled trials of interventions endorsed in primary care guidelines for the treatment of non-specific CLBP in adults. Information sources searched will include major bibliographic databases (MEDLINE, Embase, CENTRAL, CINAHL, PsycINFO and LILACS) and clinical trial registries. Our primary outcomes will be patient-reported pain ratings and treatment acceptability (all-cause discontinuation), and secondary outcomes will be functional ability, quality of life and patient/physician ratings of overall improvement. A hierarchical Bayesian class-based NMA will be performed to determine the relative effects of different classes of pharmacological (NSAIDs, opioids, paracetamol, anti-depressants, muscle relaxants) and non-pharmacological (exercise, patient education, manual therapies, psychological therapy, multidisciplinary approaches, massage, acupuncture, mindfulness) interventions and individual treatments within a class (e.g. NSAIDs: diclofenac, ibuprofen, naproxen). We will conduct risk of bias assessments and threshold analysis to assess the robustness of the findings to potential bias. We will compute the effect of different interventions relative to placebo/no treatment for both short- and long-term efficacy and acceptability.

**Discussion:**

While many factors are important in selecting an appropriate intervention for an individual patient, evidence for the analgesic effects and acceptability of a treatment are key factors in guiding this selection. Thus, this NMA will provide an important source of evidence to inform treatment decisions and future clinical guidelines.

**Systematic review registration:**

PROSPERO registry number: CRD42019138115

## Background

Low back pain is the leading cause of years lived with disability across the world [30]. It is also the second most common reason reported by patients for visiting their family doctor [24] and has an estimated lifetime prevalence of 80% [59]. The most common type of low back pain by far is the non-specific type [2], indicating the absence of an identifiable cause. While acute episodes of non-specific low back pain can improve markedly in the first 6 weeks, recent estimates suggest that pain can persist for over 12 weeks in 24–61% of cases [12]. This type of chronic low back pain (CLBP) carries an enormous economic burden both from direct (e.g. treatment) and indirect (e.g. lost work productivity) costs. In the UK, the cost to the NHS from low back pain exceeds £12 billion a year (NatCen Social [45]), with the chronic form representing the largest proportion of these costs [6]. CLBP is also associated with impaired quality of life, mobility and daily function as well as social isolation, disability and depression [46].

Because the underlying pathology of non-specific CLBP is by definition unidentified, treatment is largely focused on reducing pain symptoms, and a range of pharmacological and non-pharmacological intervention strategies are used in clinical practice [39]. A recent review of international practice guidelines [47] found that while NSAIDs and exercise were commonly recommended, the endorsement of many other treatments including opioids, antidepressants, paracetamol, muscle relaxants, spinal manipulation and acupuncture varied considerably across guidelines. The apparent uncertainty over which pool of interventions constitute the most effective options for treating non-specific CLBP suggests the need for a stronger evidence base.

Network meta-analysis (NMA) provides a powerful means of assessing multiple competing interventions by synthesising data across a network of different treatments [15]. By incorporating indirect evidence (where two treatments can be compared by assessing their performance relative to a common comparator such as placebo), the relative effects of two interventions can be evaluated even when no head-to-head trials are available. This cannot be achieved with standard pairwise meta-analysis and helps to establish a hierarchy of the best interventions for a particular condition. In addition, where there is both direct and indirect evidence, these can be combined using all the available evidence to compute the relative treatment effect.

The objective of this NMA is to assess the effectiveness and acceptability of interventions endorsed in primary care practice guidelines for the treatment of non-specific CLBP, with the aim of providing a comprehensive evidence base to inform treatment decisions. The project is called Study of Pain Interventions using Network meta-Analysis: Low-back pain (SPINAL).

## Methods/design

This protocol conforms to PRISMA-P [42] recommendations (Additional file 1) and was developed based on guidelines for systematic reviews of back pain interventions from the Cochrane Back and Neck Group [26]. Eligibility criteria were developed using the PICOS framework and are reported in detail in the following sections and summarised briefly in Table 1.

**Table 1:** Summary of PICOS eligibility criteria (“Methods/design” section lists detailed criteria)

	Inclusion criteria	Exclusion criteria
Population	Adults (≥ 18 years) with non-specific CLBP	Patient baseline pain < 4/10; radicular pain or LBP with a known cause; LBP < 12 weeks
Intervention	Primary care interventions for CLBP	Surgical or invasive interventional procedures
Comparison	A different eligible intervention or a control (placebo/sham or no intervention)	
Outcome	Pain ratings or acceptability (all cause discontinuation)	
Study type	Randomised clinical trials	

### Population

#### Inclusion criteria

We will include studies of adults (≥ 18 years) with non-specific CLBP. This is typically defined as pain without a specific known cause or pathology that persists for 12 or more weeks and that occurs below the costal margin and above the inferior gluteal folds.

Studies that simply describe low back pain as non-specific or chronic without providing detail of how this was determined will be included, provided this designation does not conflict with information elsewhere in the text (e.g. where a specific cause of LBP such as infection, cancer or fracture is listed, or where there is an obvious non-chronic symptom duration). Where it cannot be reliably determined whether LBP is specific or non-specific, we will assume non-specific as this represents the vast majority of LBP cases [47]. Where LBP duration cannot be reliably determined, we will assume LBP is acute and exclude the study as it seems likely that any chronicity would have been referred to in the text; but we will document such studies and include them as part of a sensitivity analysis if there are > 5 such studies.

#### Exclusion criteria

We will exclude studies of LBP patients with radicular pain, e.g. sciatica (or where > 10% of participants have radicular symptoms in mixed samples of patients with and without radicular pain). Radicular symptoms are typically a result of spinal nerve compromise and represent a population that may require different treatment options and who are commonly differentiated in treatment guidelines [47]. To help ensure a consistent patient population, we will exclude studies with a minimum baseline threshold for individual patient eligibility that is below 4 on a 0–10 rating, unless separate data are available for participants with baseline pain of 4 or above. We chose a threshold of 4 or above as this represents a common and established individual patient entry criterion and will ensure a homogenous sample of patients with pain of at least a moderate, clinically meaningful level [4] who are the most likely to seek treatment. If a trial does not specify individual baseline pain as an entry criterion, we will calculate *z*-scores from the sample mean baseline pain using the formula *z* = (mean baseline pain − 4.0)/SD and retain only trials where z > − 1, indicating approximately 85% of patients reporting a baseline pain of 4 or more.

Whenever we encounter trials that include both eligible and ineligible patients, we will try to determine whether data on the eligible subset can be extracted separately (e.g. in trials including both children and adults, separate the adults; in trials including both patients with and without sciatica, separate those without sciatica; in trials with baseline pain both < 4 and ≥ 4, separate those with ≥ 4 pain; and in trials with LBP duration both below and above 12 weeks, separate those with LBP ≥ 12 weeks). If the data for the eligible subset are not available from the published papers and cannot be obtained from the authors, the entire trial will be included, if the percentage of eligible patients is expected to be more than 85% (as exemplified for the baseline pain criterion above).

### Interventions

We will include interventions for the treatment of CLBP in primary care that are endorsed by any of the 15 clinical practice guidelines reviewed by Oliveira et al. [47], with the exception of herbal medicine as this is endorsed by only one guideline (and recommended against in one other guideline) and is often studied in trials of very low quality [29]. Our rationale for focusing on treatments only included in practice guidelines is that these represent the pool of intervention strategies more likely to be adopted in clinical practice and because their presence in guidelines usually indicates a higher quality evidence base [47]. Surgical and interventional pain management (e.g. spinal injections, radiofrequency denervation, deep brain and spinal cord stimulation [43]) will be excluded as these are invasive procedures that are recommended for low back only as next-line treatment in secondary or tertiary care for severe or refractory LBP where conservative primary care treatments have failed, and are not recommended in any guidelines when LBP is chronic and non-specific [47].

Both single and combined treatments are considered eligible, and medications may be fixed or flexibly dosed. For medications approved for pain, we will include only trials that use licenced dosing ranges based on European Medicines Agency guidelines. Where a drug is used off-label and no dosing guidelines exist for pain management, we will include all such trials but perform sensitivity analysis removing studies using dosages outside the approved dosing range for that drug’s approved indication.

#### Classification of interventions

Treatments will be grouped into intervention classes to allow us to compare the relative effects of intervention classes as well as individual treatments within a class, using a Bayesian hierarchical class-based NMA model [18, 19]. Grouping individual treatments into meaningful classes maximises statistical power and provides a simpler and more interpretable framework on which to ultimately inform treatment decisions (comparing each individual treatment with every other for 40 treatments, for example, would result in 780 potential comparisons). We will also perform separate analysis of pharmacological and non-pharmacological networks as described in the “Network meta-analysis” section.

Initial classifications were informed by key reviews of treatment guidelines for CLBP interventions [9, 25, 39, 46, 47, 56] and then circulated to seven members of the Lancet Low Back Pain Series Working Group (not previously known to the lead author) for evaluation and comment. We received responses from five members (see the “Acknowledgements” section), and subsequent refinements were made resulting in a final set of classifications (Table 2). Classifications are differentiated primarily by mechanisms of action, although when putative mechanisms were unclear (e.g. acupuncture) or there was uncertainty over the most appropriate classification, that treatment was listed in its own class.

**Table 2:** Intervention classes and individual treatments (generic drug names given for pharmacological agents)

Class	Examples of individual treatments
**Pharmacological**	
**Antidepressants: SNRI**	Duloxetine, desvenlafaxine, levomilnacipran, venlafaxine, milnacipran
**Antidepressants: SSRI**	Fluoxetine, fluvoxamine, paroxetine, escitalopram, citalopram, sertraline, vilazodone
**Antidepressants: tricyclic**	Amitriptyline, amoxapine, desipramine, imipramine, doxepin, clomipramine, trimipramine, protriptyline, imipramine, nortriptyline, doxepin, nortriptyline
**NSAIDs**	Ibuprofen, naproxen, sulindac, ketoprofen, tolmetin, etodolac, fenoprofen, diclofenac, flurbiprofen, piroxicam, ketorolac, Indomethacin, meloxicam, nabumetone, oxaprozin mefenamic acid, diflunisal, fenoprofen
**Opioids (strong)**	Morphine, hydromorphone, oxycodone, fentanyl, methadone, buprenorphine, diamorphine, tapentadol
**Opioids (weak)**	Codeine, hydrocodone, tramadol, pentazocine, tilidine
**Muscle relaxants: benzodiazepines**	Diazepam, estazolam, quazepam, alprazolam, chlordiazepoxide, clorazepate, lorazepam, flurazepam, clonazepam, temazepam, midazolam
**Muscle relaxants: skeletal**	Flupirtin, orphenadrine, dantrolene, carisoprodol, tizanidine, incobotulinumtoxinA, cyclobenzaprine, metaxalone, baclofen, methocarbamol, chlorzoxazone
**Paracetamol**	
**Topical agents (non-opioid)**	Diclofenac, capsaicin, lidocaine
**Non-pharmacological treatments**	
**Acupuncture**	Acupuncture, dry needling
**Exercise: non-specific**	Walking, swimming, running, stretching, aerobics
**Exercise: mind-body and bodily awareness**	Yoga, tai chi, Pilates, motor control exercise, alexander technique
**Manual therapy: spinal manipulation**	High-velocity thrust techniques at or near the end of the passive or physiologic range of motion
**Manual therapy: spinal mobilisation**	Low-grade velocity movement techniques within the patient’s range of motion and control
**Massage**	Soft tissue massage, acupressure
**Mindfulness**	Mindfulness, mindfulness-based stress reduction
**Multidisciplinary approaches**	Packages that include coordinated delivery of interventions from across different disciplinary practices/clinics (which typically consist of physical and psychological therapy, e.g. education + physiotherapy + exercise + counselling)
**Patient education: basic**	Back school (e.g. instruction on anatomy and function of the back), brief educational intervention, advice on importance of staying active, reassurance, McKenzie therapy
**Patient education: pain neuroscience**	Educational sessions that describe the neurobiology and neurophysiology of pain by the nervous system
**Psychological therapy**	CBT, operant therapy, behavioural therapy, self-regulatory therapy

A non-exhaustive list of examples of the most common interventions that comprise each class are given in Table 2. Pharmacological interventions returned by searches that are not listed in Table 2 will be classified based on MeSH and emtree headings, and non-pharmacological interventions will be classified after discussion with the review team prior to analysis with rationale for these classifications documented in the final report.

In the absence of any definitive criteria for differentiating ‘weak’ vs. ‘strong’ opioids, we followed the classifications used by Whittle et al. [57] where strong opioids are generally those with higher rates of conversion to morphine. For topical pharmacological agents, while the agents used (e.g. ibuprofen) are also often present in other classes, we nevertheless assessed this as a distinct class given the potential benefits of topical relative to systemic administration. We defined exercise therapy as ‘a series of specific movements with the aim of training or developing the body by a routine practice or as physical training to promote good physical health’ [1]. Although there are numerous meaningful ways to categorise exercise types, we decided on two basic classifications of non-specific and mind-body type approaches. However, if excessive heterogeneity is observed within each exercise type relative to other classes, we will explore sources of possible heterogeneity based on pre-defined exercise characteristics identified by Hayden et al. [33] as potentially important to efficacy (including dose/intensity, supervised vs. non-supervised, delivery type and design) and consider reclassification if necessary. Finally, as no consensus could be reached on the classification of McKenzie therapy, we provisionally classified this as education as the approach invokes components of several treatments, but we will explore the impact of this decision in a sensitivity analysis.

### Comparator

A different eligible individual treatment or a control condition (placebo/sham or no-intervention).

### Outcomes

#### Primary outcomes

1. Pain intensity, assessed with an established rating scale (e.g. 0–10 numerical rating scale or VAS) at specific time periods defined below
2. Acceptability, defined as (one minus) the proportion of patients who discontinued treatment during the trial for any reason

##### Assessment timing

The effects of different interventions on pain will be evaluated within the following distinct assessment windows: immediate (≤ 2 weeks post-randomisation), short term (> 2 weeks to ≤ 3 months), medium term (> 3 months to < 12 months) and long term (≥ 12 months). These time windows were selected based on a sample of 24 eligible articles from provisional searches. If these divisions fail to sensitively reflect the pattern of assessment timings used across studies, we may reclassify these windows prior to analysis to reflect trial practices.

As many pharmacological interventions may be more likely to be trialled for immediate and short-term outcomes, and certain non-pharmacological treatment (e.g. exercise) trials may be more likely to include long-term outcomes, separate analyses in each time window ensure that the relative efficacies of competing interventions will be evaluated in time windows appropriate for how those interventions are used. When pain ratings have been collected by the study authors at multiple time points *within* a time window, we will use the time point closest to the median for the immediate and short-term windows and the longest follow-up for the long-term follow-up window. If data are not reported at these time points (but are reported for other time points), we will make every possible attempt to retrieve these data to reduce the possibility of exaggerated treatment effects from selective reporting of the largest effects [49]. If we are unable to retrieve the preferred data, we will use outcomes at the next closest time point but conduct sensitivity analysis excluding these studies.

##### Effect sizes

Odds ratios will be computed for acceptability. If sufficient data are available, odds ratios for pain will also be computed contrasting the number of treatment responders across two interventions (or an intervention and control). A responder will be defined as a patient who demonstrates ≥ 30% and ≥ 50% reduction from baseline pain rating (we will examine both thresholds separately) reflecting ‘moderate’ and ‘substantial’ clinically important improvement according to IMMPACT recommendations [22]. When a study does not report treatment response rate, we will impute these from continuous pain ratings with an established conversion formula [28, 53], unless an excessive number of imputations are required given that this imputation assumes a normal distribution which is usually untestable.

As odds ratios can be difficult to interpret for many people, we will also present additional statistics generally perceived as more intuitive. Specifically, we will calculate risk ratios, absolute risk differences and numbers needed to treat for primary outcomes, by back transformation of the odds ratios. The baseline risk value needed for this transformation will be estimated from random-effects meta-analysis of risk from the placebo arm of placebo-controlled trials. For this purpose, we will use a subset of trials [18] judged to be representative of the overall population of chronic low back pain patients based on expert clinical input of the review team.

For pain, we will also calculate effect size as the mean difference in pain ratings across treatments, as these are expected to be reported in nearly all studies. If pain ratings are not reported on the usual 0–10 scale, they will be normalised to this scale. We will use post-treatment scores to compute effect size, unless only change from baseline scores are reported in which case we will use these. Effect sizes using either method can be legitimately pooled [13], and both produce the same effect size when study pre-treatment scores are equal across groups (as would be expected here given only randomised designs are eligible). Where we do use change from baseline scores and standard deviation(s) needed for effect size computations are not reported, they will be computed in the following priority order: first, using standard formula [5] based on the change score variance and the study pre-post correlation (or if unavailable, the average pre-post correlation across studies that report it); and second, using the average standard deviation based on studies that report it.

#### Secondary outcomes

Based on recommendations for a core outcome set (COS) in non-specific low back pain [8], we also included the following outcomes and associated recommended assessment measures:

1. Physical functioning (PF), assessed with the Oswestry Disability Index 2.1a or Roland-Morris Disability Questionnaire (the two recommended COS measures and the most commonly used in trials). If a study does not employ either scale, we will include any of the following: Quebec Back Pain Disability Scale, BPI-PI, MPI-PI, SF-36-PF, PROMIS-PF, CLBPDQ, LBPRS-DI, and ODI 1.0 as there is evidence of their validity as assessments of PF [8]
2. Health-related quality of life, assessed with the Short-Form Health Survey (SF-12/ SF-36) or PROMIS-GH-10.
3. Patient or physician ratings of overall improvement.

As all secondary outcomes are assessed on a continuous measure, we will use the mean difference as the effect size. If an outcome is assessed by multiple different scales, we will use the most common scale and convert scores from any other scales to the same metric if an established mapping algorithm exists. If this results in a low number of available studies (e.g. < 60% of the total studies reporting that outcome), to maximise data inclusion, we will standardise all scales for that outcome and use the *standardised* mean difference, provided that an inspection of the domain of the scales suggests the scales can be meaningfully combined. We will conduct sensitivity analysis in all instances where scales have been combined.

#### Outcomes with missing data

Where missing participant data is present, studies may report analysis on only the subset of patients who adhered to the intervention (per-protocol) or on all participants who were assigned to the intervention at the start of the trial (intention-to-treat) after missing data has been imputed (e.g. using last observation carried forward). If both per-protocol and intention-to-treat analyses are reported, we will prioritise intention-to-treat data [54]. In all instances, we will report whether analysis was conducted on data that were complete, complete after imputation or incomplete, and we will examine and report any material differences in results across these types. When primary outcomes are missing, an effort will be made to contact authors to obtain data.

#### Study designs

Only randomised controlled trials comparing an active intervention with another eligible intervention or control will be included. Randomisation can be at the individual or group level, and both parallel group and crossover designs will be included. For crossover designs, only data from the first trial period will be extracted to eliminate any possibility of carryover effects.

#### Language

No language restrictions will be initially applied, although studies for which adequate translation cannot be obtained will be considered potentially eligible and described in the final report but will not be included in the meta-analysis.

### Information sources

We will search for published RCTs indexed in the following databases by the final search date: MEDLINE (1946-), MEDLINE In-Process, EMBASE (1974-), CENTRAL, CINAHL (1937-), LILACS (1982-) and PsycINFO (1967-). We will also search for published, unpublished and ongoing trials in clinical trial registries ClinicalTrials.gov and WHO International Clinical Trials Registry Platform (ICTRP). We will complement published data with results reported in these trial registries. We will additionally search the websites of drug regulatory bodies of the FDA (USA), MHRA (UK) and EMA (Europe). It is important to include unpublished data, since the well-known bias towards publication of significant findings can, when relying on published literature alone, lead to an overestimation of treatment effects and an underestimation of adverse effects [21]. The search strategy will be augmented through hand searching of relevant reviews and of the reference lists of included articles for additional studies.

For unpublished clinical trials, if a study is listed as ongoing and ≥ 1 year has elapsed since registration, we will attempt to establish whether the listed trial status is current. If it emerges that such trials have in fact been completed or terminated, we will attempt to obtain data from: (a) the trial registry, (b) study authors, (c) drug regulatory agency websites and (d) OpenTrials (which while still in its preliminary stages can provide a wide range of unpublished evidence including regulatory documents, clinical study reports and protocols). Where possible, the same sources will be approached when a trial has been published but key primary outcomes are not reported or reported only partially in the journal publication.

### Search strategy

The search strategy was informed by PICOS criteria and will be comprised of three groups of terms relating to (1) randomised trials, (2) CLBP and (3) interventions. Search terms will be combined with a Boolean “AND” and consist of both controlled subject headings (where provided by the database) and free-text keywords in titles and abstracts.

Randomised trials will be identified using highly sensitive search filters validated for each database [23, 31, 41, 58] and CLBP studies identified using search terms suggested by Furlan et al. [26]. For identifying treatments, we will employ subject headings for intervention trials and an extensive list of keywords for specific interventions from clinical practice guidelines [25, 46, 47] and relevant Cochrane Reviews (https://back.cochrane.org/our-reviews).

Search strings were reviewed and approved by a healthcare information specialist at the University of Greenwich (see Additional file 2 for the draft MEDLINE example).

### Study selection

Records returned by initial searches will be screened for relevancy in two stages. First, the titles and abstracts of each record will be independently screened by two members of the review team, who will exclude studies not meeting the eligibility criteria. The online software Rayyan [48] will be used to facilitate first stage screening by highlighting keywords relating to inclusion and exclusion criteria. Second, the full-text of the remaining articles will be screened by the same two reviewers, who will retain for inclusion in the NMA only those that meet the eligibility criteria. Disagreements at any stage will be resolved through discussion or, if not resolved, with a third member of the review team.

### Data extraction

Data from each study will be extracted by one member of the review team and checked for accuracy by a senior member of the review team, with sets of studies distributed across a pool of reviewers. We will use a standardised excel coding form adapted from our previous work, with explanatory notes provided on how coding should be performed for each variable to ensure consistency across coders. If there are missing method data or missing outcome data, the corresponding author will be contacted via e-mail with one additional reminder email sent within 3 weeks if no response is received. Subsequently, other authors will be contacted. If no response is received before analysis is conducted, the study will be excluded from the NMA but the basic study findings will be described in a separate section of the final report. When data are identified as being published across multiple sources, we will prioritise extraction from the most complete data sources. Where these sources include both published and unpublished data, we will extract both but prioritise published data in the analysis as this has been subject to peer-review, but conduct sensitivity analysis including both published and unpublished data.

When available study data do not allow computation of effect sizes using standard formula (e.g. based on means and SDs) we will (a) extract other statistics (e.g. *F*, *p*, *t*) that allow effect sizes to be computed using alternative formula [11]; (b) contact study authors for data, and (c) for missing SDs, use the pooled SD from other studies [27] or external data. Finally, where a pain rating scale assesses not only average pain, but least and worst pain over the previous period (as in the Brief Pain Inventory), we will use only average pain ratings.

### Data items

Study information extracted will include (1) study identifiers (e.g. title, authors, publication date); (2) study characteristics (e.g. trial design, source of financial support, trial size, study location); (3) participant characteristics (e.g. mean sample age, male/female ratio, SES, pain duration, severity and current or previous treatments); (4) intervention details (e.g. type and class of treatment, intervention details, duration, dosage, delivery method); and (5) outcome data (including assessment used, timing, missing data details).

### Robustness of findings and risk of bias

Risk of bias will be assessed for all studies using the revised Cochrane Risk of Bias (RoB) tool (RoB 2.0 [54]). Assessments will be carried out independently by two reviewers, with any disagreement resolved by discussion or, if needed, consultation with a third reviewer. We will also collect additional measures of bias (see “Meta-regression and sensitivity analysis”) and examine their potential influence in meta-regression.

We will conduct threshold analysis [7, 50] to quantify the level of bias that would have to be present in the estimated treatment effect to have resulted in a major change in treatment ranking (such as a change in the order of the highest ranked interventions). If the magnitude of such potential bias is implausible, then conclusions on the ‘best’ treatments are more robust. If the level of bias needed to overturn treatment decisions *is* plausible, then we will closely examine RoB scores for that treatment as well as relevant external work to determine whether such bias is likely to be present to help evaluate our confidence in the findings.

An alternate method for assessing robustness is Salanti’s [51] GRADE for NMA extension, implemented using the CINeMA web application. This estimates overall RoB for a treatment comparison by aggregating individual study RoB scores after weighting each score based on a study’s contribution to the overall treatment effect size. For the proposed NMA, however, we chose threshold analysis as we will employ a Bayesian analysis (CINeMA currently applies frequentist weights), and threshold analysis is more suited to directly informing treatment decisions [50].

## Data synthesis and analysis

We will provide a descriptive table summarising the key characteristics of each eligible study, including interventions, patient populations and trial characteristics. A network diagram will show which intervention classes were compared, with larger network nodes indicating a greater number of patients and thicker connecting lines between nodes indicating a greater number of trials.

### Consistency assumption

A key assumption of NMA is that each participant should be equally likely to have received any of the treatments in the network. If this assumption holds, a key consequence is that there should be no systematic differences in effect modifiers (such as important patient characteristics) across different sets of treatment comparisons that might otherwise explain apparent intervention differences [10].

As described in the “Population” section, we will ensure similarity by restricting patient populations to those with non-specific LBP that is chronic only and who report a moderate or greater level of pain. We will also qualitatively assess the clinical similarity of populations across different treatment comparisons on potentially important factors, such as age, sex, baseline pain severity and CLBP duration [3, 32, 40], and present this in a summary table. Statistical tests of consistency we will employ are described in the “Assessment of consistency” and the “Assessment of within-comparison heterogeneity” sections. One common concern with comparing pharmacological and non-pharmacological interventions in general is that one class of intervention is administered as a first-line treatment and the other is given to treatment-resistant cases for whom previous interventions have failed. Because we are examining chronic LBP, however, treatment failure would have been likely for all patients during the acute phase of their LBP in order for chronic LBP to develop.

### Network meta-analysis

We will conduct a Bayesian NMA to estimate relative treatment effects based on a synthesis of direct (head-to-head trials) and indirect evidence (where two treatments are compared indirectly via a common comparator). We will use a class-based hierarchical model [18] to estimate the relative effects of different treatment classes (e.g. NSAIDs, opioids) and of individual treatments within a class (e.g. ibuprofen, aspirin, diclofenac). Pharmacological and non-pharmacological studies may differ in patient and study characteristics and type of biases that may exist. As such, we will conduct separate analyses of these two networks along with an analysis of the whole network (providing head-to-head comparisons of pharmacological and non-pharmacological interventions are available) to see if these two approaches yield similar results.

The relative effectiveness of different treatments will be modelled as a function of their performance relative to a placebo reference treatment. This will be presented as a forest plot for class effects and in table form for class and individual effects. Mean ranks with their 95% credible intervals and SUCRA (a simple transformation of the mean rank) will be used to provide a hierarchy of the best treatments.

#### Estimation details

Model parameters will be estimated in WinBUGS using Markov Chain Monte Carlo simulation. Posterior distributions will be derived from binomial (binary outcomes) and normal (continuous) likelihood functions using vague prior distributions. For within-treatment study variability, we will assume a common heterogeneity standard deviation and use a partially informative uniform prior with an upper bound limit based on the outcome scale used (e.g. *U*(0, 10) for pain ratings). For within-class variability (of treatments), we will use a uniform prior distribution estimated separately for each class. However, for classes with only a few elements, decisions will be made on whether the within-class variance estimates can be shared across similar classes (e.g. SNRI and SSRI classes). For other parameters, we will use wide non-informative normal priors. We will examine Gelman-Rubin trace plots to check that multiple chains achieve convergence during the burn-in period, and base our estimates on 50,000 or more subsequent iterations to ensure MC estimator error is less than 5% of the standard deviation for the treatment effect and heterogeneity parameters. With respect to multi-arm trials, the correlation between multiple treatment comparisons within these trials is naturally accounted for within the Bayesian framework.

The choice between a random-effects (RE) and fixed-effect (FE) model will be informed by a comparison of Deviance Information Criteria (DIC) model fit statistics. If the DIC for the RE model is at least 3 units lower (with lower values indicating better fit) [18], we will use a RE model. If the models are otherwise similar, we will choose the more parsimonious FE model provided there is no excessive study heterogeneity from separate pairwise analysis.

#### Assessment of consistency

We will assess whether there is consistency of direct and indirect evidence globally across the whole network (which is a natural consequence of the similarity assumption) using the unrelated mean effects model [17]. If evidence of inconsistency is found, we will use a node-splitting approach [16] to identify possible areas of local inconsistency and, if sufficient data exist, run network meta-regression to examine whether inconsistency (and study heterogeneity) is resolved by a consideration of differences in clinical variables (the “Consistency assumption” section).

In the event of minor unresolved inconsistency, we will proceed with NMA but advise caution in the interpretation of results for comparisons where there are material differences between direct and indirect estimates. If there is evidence of substantive inconsistency, we will consider excluding network nodes.

#### Assessment of within-comparison heterogeneity

Study heterogeneity within each treatment comparison will be examined with forest plots from pairwise meta-analysis for an initial visual assessment (and these will be used to alert us to potential outliers). We will also compute *I*^2^, which indicates the proportion of overall variance in effect sizes due to genuine heterogeneity. *I*^2^ > 60% can indicate a moderate or greater variation in study effect sizes [34] and will be explored with meta-regression. We will also compute Cochran’s *Q* with *p* < .10 used to indicate possible presence of heterogeneity and tau-squared to provide an estimate of effect size heterogeneity for different comparisons

### Meta-regression and sensitivity analysis

Given sufficient data, we will use network meta-regression to explore whether inconsistency/heterogeneity and group differences in the two primary outcomes is influenced by potential biases such as industry sponsorship, performance in less (vs. more) developed countries [14], risk of bias scores, novel agent effects [52] and researcher allegiance to the study intervention [20]. Two members of the review team will independently assess researcher allegiance (with any disagreement resolved by consensus) using a checklist developed and piloted for the current study (Additional file 3) based on the modified reprint method [44]. We will also include effect size derivation method (post vs. change scores) as a dummy-coded covariate to check that effect sizes from both methods are similar.

We will produce treatment-control comparison-adjusted funnel plots to explore possible publication bias, and if bias is suspected explore this by including sample size as a covariate. We will also perform a test of excess significance [36] which is applied to data aggregated across the whole network of interventions (thus offering higher statistical power than pairwise tests) to assess whether there is an excess of statistically significant findings.

We will also assess the robustness of the findings to various decisions by performing sensitivity analyses including removing studies (a) with high risk of bias, (b) where imputations have been performed, (c) where we assumed LBP was non-specific when this could not be definitively determined (the “Population”section), and (d) where very high/low dosages were used for off label medications. In addition, we will rerun the analysis after reclassifying McKenzie therapy into mind-body awareness exercises based on feedback from the Lancet LBP working group.

### Unit of analysis issues

For trials that use cluster randomisation without adjusting standard errors for the study’s design effect [35], we will apply this adjustment ourselves. As intra-class correlations needed to make this correction are seldom reported, we will use values obtained from external literature for the outcome examined (or if these are not available use a single plausible value and examine the impact of varying this value in sensitivity analysis).

## Discussion

The results from this NMA will provide an important evidence base for clinicians to inform treatment decisions by providing a comparative assessment of a wide range of interventions [55]. This will help efforts to develop a precision medicine approach to the treatment for non-specific chronic low back pain, which can be used in everyday clinical settings. While there are numerous factors that must be considered in treatment decisions, such as cost-effectiveness, individual patient suitability and patient preferences [37], reliable information on the pain-relieving effects and acceptability of a treatment as well as an assessment of how bias-free these results might be are fundamental points in guiding these decisions.

Given the sheer scale of the burden of chronic low back pain, we expect the results of the NMA to be of considerable interest to clinicians, academics, guideline developers and policymakers [38] and we will disseminate the findings widely through academic publications, conference presentations and communication with healthcare providers.

## Supplementary information

**Additional file 1.** PRISMA-P checklist.

**Additional file 2.** MEDLINE search string.

**Additional file 3.** Researcher allegiance.

## Data Availability

Not applicable

## Abbreviations

CINeMA: Confidence in network meta-analysis
COS: Core outcome set
CLBP: Chronic low back pain
FDA: Food and Drug Administration
GRADE: Grading of Recommendations, Assessment, Development and Evaluation
IMMPACT: Initiative on Methods, Measurement, and Pain Assessment in Clinical Trials
LBP: Low back pain
NMA: Network meta-analysis
NSAIDs: Non-steroidal anti-inflammatory drugs
PF: Physical functioning
PICOS: Population, intervention, comparator, outcomes, study design
PRISMA-P: Preferred Reporting Items for Systematic review and Meta-Analysis Protocols
RoB: Risk of bias
SNRI: Serotonin–norepinephrine reuptake inhibitor
SSRI: Selective serotonin reuptake inhibitor
SUCRA: Surface Under the Cumulative RAnking curve
WHO: World Health Organization
