# Supplementary material for: Efficacy and acceptability of pharmacological and non-pharmacological interventions for non-specific chronic low back pain: a protocol for a systematic review and network meta-analysis
Source: Syst Rev. 2020 Jun 5;9:130. doi: 10.1186/s13643-020-01398-3 (PMC7275431; doi:10.1186/s13643-020-01398-3)
Supplement: Supplementary file 2 — Additional file 2. MEDLINE search string. [file 13643_2020_1398_MOESM2_ESM.docx]

**Additional File 2**

**Search string for MEDLINE (OVID)**

1 Randomized controlled trial.pt.

2 Controlled clinical trial.pt.

3 randomi?ed.ab.

4 placebo.ab.

5 drug therapy.fs.

6 randomly.ab.

7 trial.ab.

8 groups.ab.

9 or/1-8

10 animals.sh. not (humans.sh. and animals.sh.)

11 9 not 10

12 exp Low Back Pain/

13 (dorsalgia or backache or lumbago or coccydynia).tw.

14 (back ADJ1 pain).tw.

15 (lumbar ADJ1 pain).tw

16 (coccyx ADJ1 pain).tw

17 or/12-16

18 exp pain management/

19 exp mind-body therapies/

20 exp patient education as topic/

21 exp exercise/

22 exp exercise therapy/

23 exp cognitive behavioral therapy/

24 exp acupuncture/

25 exp mindfulness/

26 exp "physical and rehabilitation medicine"/

27 exp musculoskeletal manipulations/

28 (mind?body or tai?ji or tai?chi or taiji or yoga or mind?body or musculoskeletal or manipulation$ or soft tissue therapy or acupressure or massage or rehabilitation or spinal adjustment or spinal mobilisation or acupuncture or mindfulness or exercise movement or exercise or running or swimming or walking or stretching or physical activity or physical conditioning or remain active or aquarobics or cognitive behavio?ral therapy or behavio?r therapy or psychological therapy or "acceptance and commitment" therapy or rehabilitation or acupuncture or progressive relaxation or operant therapy or psychological therap$ or pilates or motor control exercise or mce or education or back school$).ti,ab.

29 or/18-28

30 exp analgesics/

31 exp acetaminophen/

32 (acetaminophen or acamol or acephen or acetaco or acetamidophenol or acetaminophen or acetominophen or algotropyl or anacin 3 or anacin-3 or anacin3 or datril or hydroxyacetanilide or "n-(4-hydroxyphenyl)acetanilide" or n-acetyl-p-aminophenol or panadol or paracetamol or tylenol or p-acetamidophenol or p-hydroxyacetanilide).mp.

33 or/30-32

34 exp Anti-Inflammatory Agents, Non-Steroidal/

35 aspirin.mp. or exp Aspirin/

36 etodolac.mp. or exp Etodolac/

37 diclofenac.mp. or exp Diclofenac/

38 sulindac.mp. or exp Sulindac/

39 (indometacin or indomethacin).mp. or exp Indomethacin/

40 piroxicam.mp. or exp Piroxicam/

41 fenoprofen.mp. or exp Fenoprofen/

42 flurbiprofen.mp. or exp Flurbiprofen/

43 ibuprofen.mp. or exp Ibuprofen/

44 ketoprofen.mp. or exp Ketoprofen/

45 naproxen.mp. or exp Naproxen/

46 diflunisal.mp. or exp Diflunisal/

47 metamizol.mp. or exp Dipyrone/

48 phenylbutazone.mp. or exp Phenylbutazone/

49 phenazone.mp. or exp Antipyrine/

50 exp cyclooxygenase inhibitors/ or exp cyclooxygenase 2 inhibitors/

51 exp Meclofenamic Acid/

52 piroxicam.mp. or exp Piroxicam/

53 tolmetin.mp. or exp Tolmetin/

54 (nsaids or non?steroidal anti?inflammat$ or acetylsalicyl$ or carbasalate calcium or aceclofenac or alclofenac or meloxicam or dexibuprofen or dexketoprofen or tiapro$ or propyphenazone or celecoxib or etoricoxib or nabumeton or parecoxib or ((cyclooxygenase or cyclo-oxygenase) adj3 inhibitor*) or rofecoxib or valdecoxib or lumiracoxib or vioxx or celebrex or bextra or prexige or arcoxia or floctafenine or meclofenamate or oxaprozin or tenoxicam).mp.

55 or/34-54

56 exp analgesics, opioid/

57 (alfentanil or alphaprodine or buprenorphine or butorphanol or codeine or dextromoramide or dextropropoxyphene or dihydromorphine or diphenoxylate or ethylketocyclazocine or ethylmorphine or etorphine or fentanyl or hydrocodone or hydromorphone or levorphanol or meperidine or meptazinol or methadone or methadyl acetate or morphine or nalbuphine or opiate alkaloids or opium or oxycodone or oxymorphone or pentazocine or phenazocine or phenoperidine or pirinitramide or promedol or remifentanil or sufentanil or tapentadol or tilidine or tramadol).mp.

58 or/56-57

59 Antidepressive Agents.mp or antidepress$.mp. or exp Antidepressive Agents/

60 (aripiprazole or benactyzine or clorgyline or deanol or desvenlafaxine succinate or duloxetine or iproniazid or isocarboxazid or levomilnacipran or mirtazapine or moclobemide or nialamide or phenelzine or pizotyline or reboxetine or rolipram or selegiline or sertraline or tranylcypromine or vilazodone or vortioxetine or desvenlafaxine or lithium or vilazodone hydrochloride or amitriptyline or amoxapine or clomipramine or desipramine or dothiepin or doxepin or imipramine or iprindole or lofepramine or nortriptyline or opipramol or protriptyline or trimipramine).mp.

61 or/59-60

62 Neuromuscular Agents.mp or muscle relaxant?.mp.or exp Neuromuscular Agents/

63 (chlormezanone or chlorphenesin or chlorzoxazone or dantrolene or diazepam or medazepam or mephenesin or meprobamate or methocarbamol or orphenadrine or quinine or tolperisone or xylazine or zoxazolamine or botulinum toxins or amifampridine or baclofen or carisoprodol or doloteffin).mp.

64 or/62-63

65 benzodiazepines.mp. or exp benzodiazepines/

66 (tetrazepam or diazepam or alprazolam or anthramycin or bromazepam or clonazepam or devazepide or diazepam or diazemuls or faustan or valium or seduxen or sibazon or stesolid or apaurin or relanium or flumazenil or flunitrazepam or flurazepam or lorazepam or nitrazepam or oxazepam or pirenzepine or prazepam or temazepam).mp.

67 or/65-66

68 (prednisone or cortisone or dexamethasone or capsaicin or lidocaine or dmso or dimethyl sulphoxide).mp.

69 33 or 55 or 58 or 61 or 64 or 67 or 68

70 29 or 69

71 11 AND 17 AND 70
