# Supplementary material for: Efficacy and acceptability of pharmacological and non-pharmacological interventions for non-specific chronic low back pain: a protocol for a systematic review and network meta-analysis
Source: Syst Rev. 2020 Jun 5;9:130. doi: 10.1186/s13643-020-01398-3 (PMC7275431; doi:10.1186/s13643-020-01398-3)
Supplement: Supplementary file 3 — Additional file 3. Researcher allegiance. [file 13643_2020_1398_MOESM3_ESM.docx]

**Additional File 3**

**Researcher Allegiance (RA) Bias**

For the proposed NMA, we have developed a new RA scale (see Table 1) that uses a modified reprint method and which was adapted from several existing scales (see Yoder et al., 2019). These adaptations were performed to: (1) ensure scale items were applicable to all types of intervention defined in our protocol (several previous scales are worded specifically for psychotherapy interventions); (2) exclude items where a high RA rating is as likely to result from significant study findings or a motivation to publish (e.g. “the authors’ review of previous evidence favours the treatment”) as much as any actual pre-existing treatment allegiance (Leykin and DeRubeis, 2009), and; (3) to employ greater use of external information (which may be especially important when assessing unpublished trials) that is not contained in the study report.

After initial development by one author (TT), the scale was refined following pilot testing from two authors (DP, SW) on a representative sample of ten eligible studies. These refinements primarily involved the elaboration of explanatory scoring notes and the use of a continuous total score (0-6), as the original binary total score (1= if *any* of the six items are endorsed, 0 = if no items are endorsed) produced a ceiling effect.

It should be noted that it is probably more accurate to describe any RA scale as providing a measure of the *potential* for RA bias. While the items assess factors that could plausibly lead to a biased exaggeration of any positive effects of a treatment (Dragioti et al., 2015b), it is difficult to measure whether actual bias is present (e.g. whether a grant from a sponsor connected to the treatment does result in actual bias). Nevertheless, if an association between RA scores and treatment efficacy is found in the analysis, then this does suggest further investigation and cautious interpretation of the findings is warranted (Dragioti et al., 2015a).

**References**

Dragioti, E., Dimoliatis, I., & Evangelou, E. (2015a). Disclosure of researcher allegiance in meta-analyses and randomised controlled trials of psychotherapy: a systematic appraisal. *BMJ Open*, *5*(6), e007206. doi:10.1136/bmjopen-2014-007206

Dragioti, E., Dimoliatis, I., Fountoulakis, K. N., & Evangelou, E. (2015b). A systematic appraisal of allegiance effect in randomized controlled trials of psychotherapy. *Ann Gen Psychiatry*, *14*, 25. doi:10.1186/s12991-015-0063-1

Leykin, Y., & DeRubeis, R. J. (2009). Allegiance in psychotherapy outcome research: Separating association from bias. *Clinical Psychology: Science and Practice*, *16*(1), 54-65. Retrieved from http://www.dgapractice.com/documents/AlliegianceinPsychotherapyOutcomeResearch.pdf

Yoder, W. R., Karyotaki, E., Cristea, I. A., van Duin, D., & Cuijpers, P. (2019). Researcher allegiance in research on psychosocial interventions: meta-research study protocol and pilot study. *BMJ Open*, *9*(2), e024622. doi:10.1136/bmjopen-2018-024622

**Table 1. Researcher Allegiance Checklist**

|  | **Explanatory notes** | **Usual detection method** |
| --- | --- | --- |
| *1. Use of the intervention in clinical practice* | Score 1 if the lead or corresponding author appears to be active in the use of the intervention* in a clinical setting, as a consultant or in any capacity as a paid service | Typically an internet search |
| *2. Relevant sponsorship/funding* | Score 1 if the funder is likely to have a vested interested in the outcome (such as a pharmaceutical company or a retailer of an intervention product). Funding from a public body (e.g. NiH) or no funding would typically be scored as 0. | From the article (e.g. in Conflict of Interest / Funding section) |
| *3. Relevant research group membership* | Score 1 if the lead or corresponding author appears to belong to a research group specific to the intervention* examined (and thus where there is a vested academic benefit even if not a financial one) e.g. ‘mindfulness for pain management group’). Where there is no detectable research group membership or membership of a general research group (e.g. Pain Management in Chronic Illness group ) this would typically be scored 0. | From the study report or an internet search |
| *4. Contribution to the development of the intervention or the underlying aetiological model* | Score 1 if the lead or corresponding author can be identified as contributing to the development of the aetiological model underlying the intervention or to the development of the intervention itself in its original or adapted form (e.g. of a novel psychosocial approach to pain management) | Typically referred to in the article |
| *5. Previous advocacy of the intervention in any form* | Score 1 if there is any evidence that the lead or corresponding author has advocated the use of the specific intervention* in any form | Probably from the study report’s reference list or an internet search |
| *6. Other credible evidence for allegiance* | Score 1 if there is credible other evidence for allegiance. Note a score of 1 requires agreement from at least two other members of the study team |  |

**Scoring**

Individual item scores are summated to give an overall RA rating of 0-6.

*For head-to-head trials (with no control group), items will be scored 1 if there is evidence of RA for the *treatment with the most favourable effect*. Where head-to-head trials also incorporate a control group comparator, scoring will be conducted separately for each intervention.
